# Salvage nivolumab and ipilimumab after prior anti‐PD‐1/PD‐L1 therapy in metastatic renal cell carcinoma: A meta‐analysis

**DOI:** 10.1002/cam4.4587

**Published:** 2022-02-09

**Authors:** Yuanquan Yang, Sherry V. Mori, Mingjia Li, Megan Hinkley, Anish B. Parikh, Katharine A. Collier, Abdul Miah, Ming Yin

**Affiliations:** ^1^ Division of Medical Oncology The Ohio State University College of Medicine Columbus Ohio USA

**Keywords:** immune checkpoint inhibitor, ipilimumab, meta‐analysis, nivolumab, renal cell carcinoma, salvage treatment

## Abstract

**Background:**

Salvage nivolumab and ipilimumab after prior anti‐PD‐1/PD‐L1 therapy is frequently used off‐label for clear cell metastatic renal cell carcinoma (mRCC). However, limited data are available to guide such therapy. We performed a meta‐analysis to characterize further the safety and efficacy of salvage nivolumab and ipilimumab.

**Methods:**

We conducted a systematic review in accordance with PRISMA. Studies of salvage nivolumab and ipilimumab in patients with mRCC published in English before June 1, 2021 were included. We also included patients treated at the Ohio State University from 2012 to 2020 through a retrospective chart review. The included studies were further stratified into adaptive and standard groups based on their designs. We calculated objective response rate (ORR) and adverse events (AEs) via pooled data and quantitative synthesis using the Stata metaprop procedure. A conservative random effect model was used to combine values.

**Results:**

A total of 7 studies and 310 patients were included. Salvage nivolumab and ipilimumab had an ORR of 14% (95% CI, 0.09–0.21) and median progression‐free survival ranged between 3.7 and 5.5 months. Four out of the seven studies were standard design, whereas the other three studies were adaptive. The ORR was numerically higher in the standard group compared with the adaptive group (21% and 9–10%, respectively). The responses to salvage nivolumab and ipilimumab did not correlate with the initial anti‐PD‐1/PD‐L1 responses (odds ratio = 1.45; *p* = 0.5). Grade ≥3 AEs occurred in 26% of the patients (95% CI, 0.19–0.33). There were no new safety signals observed in this study.

**Conclusion:**

Salvage nivolumab and ipilimumab demonstrated moderate antitumor activity and a manageable safety profile in patients with mRCC who had prior anti‐PD‐1/PD‐L1 therapy.

**Implication for Practice:**

Patients with metastatic renal cell carcinoma have limited treatment options after progressive disease on anti‐PD‐1/PD‐L1 therapy. The role of salvage nivolumab and ipilimumab in this patient population is poorly defined. The studies on this highly important and clinically relevant topic are limited by small sample sizes. The results from our meta‐analysis suggest that nivolumab and ipilimumab are feasible in the salvage setting with moderate efficacy and acceptable toxicity profile. The response rates differ with different treatment designs. This information will be beneficial to guide clinical decision‐making and accurately estimating toxicity.

## Graphical abstract

The role of salvage nivolumab and ipilimumab after progressive disease on anti‐PD‐1/PD‐L1 therapy is poorly defined. The studies on this highly important and clinically relevant topic are limited by small sample sizes. The results from our meta‐analysis suggest that nivolumab and ipilimumab are feasible in the salvage setting with moderate efficacy and acceptable toxicity profile. The response rates differ with different treatment designs.

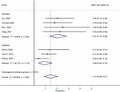

## 1 INTRODUCTION

Vascular endothelial growth factor receptor (VEGFR) tyrosine kinase inhibitors (TKI) and immune checkpoint inhibitors (ICI) targeting the PD‐1/PD‐L1 axis are the cornerstones for clear cell metastatic renal cell carcinoma (mRCC) management.^1^, ^2^ VEGFR‐TKIs (e.g., sunitinib) alone, dual ICI (nivolumab and ipilimumab), and TKI–ICI combinations (such as axitinib and pembrolizumab) are all FDA‐approved as front‐line options for mRCC.^3^, ^4^, ^5^ Clinicians often make treatment decisions based on International mRCC Database Consortium (IMDC) risk, disease burden, and comorbid conditions. Nivolumab (an anti‐PD‐1 antibody) monotherapy is the preferred second‐line option after prior VEGFR‐TKI failure.^6^ After progression on anti‐PD‐1/PD‐L1 therapy, VEGFR‐TKI is the treatment of choice. However, the response to VEGFR‐TKI is often short‐lived. Therefore, there is an unmet need for durable salvage therapies.

Nivolumab plus the anti‐CTLA‐4 antibody ipilimumab is approved based on superior overall survival in patients with IMDC intermediate‐/poor‐risk diseases compared with sunitinib.^7^ In an updated report, 31% of patients had durable responses after a minimal 4‐year follow‐up.^8^ Salvage nivolumab and ipilimumab are often used off‐label in patients with prior exposure to anti‐PD‐1/PD‐L1 therapy who are ipilimumab naive. However, the benefit and safety of such an approach have not been well characterized. There is limited data, and small study sample sizes restrict the validity.^9^, ^10^, ^11^, ^12^, ^13^, ^14^ For example, the majority of studies have a sample size of <50, while the reported objective response rates (ORR) range from 4% to 25%. Based on such numbers, the efficacy of salvage use of nivolumab plus ipilimumab can be little (4%) or moderately significant (25%). What is more, different study designs were used to evaluate salvage treatment of nivolumab and ipilimumab, making it difficult to compare results from individual studies. For example, some studies switched to nivolumab and ipilimumab after other treatment failures,^9^, ^10^, ^11^ whereas others added ipilimumab to nivolumab after suboptimal response to nivolumab monotherapy.^12^, ^13^, ^14^ Hence, we performed a meta‐analysis to further characterize the safety and efficacy of salvage nivolumab and ipilimumab after prior anti‐PD‐1/PD‐L1 therapy in mRCC, either as a whole or by different designs.

## 2 METHODS

### 2.1 Literature search

We conducted this systematic review by following the guidelines of PRISMA. We searched Medline and Scopus databases as well as conference abstracts published in English before June 1, 2021, using the keywords “renal cell carcinoma” or “cancer” or “tumor,” “nivolumab” and “ipilimumab,” “re‐challenge” or “salvage” or “refractory.” We included published studies or abstracts that reported treatment efficacy and toxicity of nivolumab and ipilmumab in mRCC patients who had prior exposure to anti‐PD‐1/PD‐L1 therapy. Studies of first‐line treatment of nivolumab and ipilimumab, performed in nonrenal cancer, or lack of anti‐PD‐1/PD‐L1 therapy exposure, or nonoriginal investigation, were excluded. Two reviewers independently screened the literature unblindly and included six studies reporting salvage nivolumab and ipilimumab outcomes of mRCC patients (Figure S1). We added additional information from 27 patients with mRCC who received salvage nivolumab and ipilimumab at the Ohio State University (OSU) after anti‐PD‐1/PD‐L1 therapy from 2012 to 2020 (Table S1). The study was approved by Institutional Review Board, and the patient consent was waived. The local cohort was referred to as Yang (2021) in the manuscript. A total of seven studies were included in this meta‐analysis (Table 1).

**TABLE 1:** Study characteristics and efficacy results

Study	IMDC	Design	Total *N*	Salvage Nivo/Ipi ORR		Median PFS
			(*n* = OR)	Pooled analysis	Quan synthesis	(*m*, 95% CI)
**Standard**						
Gul, 2020	All	retrospective	44 (9)		0.2 (0.1–0.35)	4 (0.8–19)
Choueiri, 2020	All	prospective	39 (7)		0.18 (0.08–0.34)	3.7 (2.2–7.3)
Ravi, 2020	All	retrospective	20 (5)		0.25 (0.09–0.49)	NA
Yang, 2021	All	retrospective	22 (5)		0.23 (0.08–0.45)	4 (2.4–6.2)
**Subtotal**			**125 (26)**	**0.21 (0.14–0.29)**	**0.21 (0.14–0.28)**	
**Adaptive**						
Atkins, 2020	All	prospective	30 (4)		0.13 (0.04–0.31)	NA
Grimm, 2019	Int/high	prospective	102 (12)		0.12 (0.06–0.20)	1L 5.5 (NA), 2L 3.7 (NA)^a^
McKay, 2020	All	prospective	53 (2)		0.04 (0–0.13)	4.7 (2.7–8.3)
**Subtotal**			**185 (18)**	**0.10 (0.06–0.15)**	**0.09 (0.04–0.16)**	
**All total**			**310 (44)**	**0.14 (0.11–0.19)**	**0.14 (0.09–0.21)**	

a Median PFS in patients enrolled with 1) no prior therapy (1L) or 2) one line of prior VEGFR tyrosine kinase inhibitor (2L).

### 2.2 Data extraction

We extracted the following information from each study: author, year of publication, study design (retrospective vs. prospective), risk stratification by IMDC, sample size, number of objective responses (OR), median progression‐free survival (PFS), and toxicity profiles. Since the study by Ravi et al. reported the outcomes of multiple different immunotherapy regimens, we reconstructed OR data of salvage nivolumab and ipilimumab from the supplementary data.

### 2.3 OSU data analysis

Treatment response was defined as best response by complete response (CR) and partial response (PR) based on RECIST 1.1 criteria. PFS was defined as the time of nivolumab and ipilimumab therapy initiation to the date of radiographic disease progression or death from any cause. For patients who had not yet progressed but were switched to another therapy (e.g., due to toxicity), PFS was censored at the date of the last evaluable tumor assessment prior to the treatment change. Safety and tolerability were determined by related descriptions in the chart review and were assessed for grade ≥3 treatment‐related adverse events (AE) based on the Common Terminology Criteria for Adverse Events Version 5.0.

### 2.4 Quantitative synthesis and bias assessment

We assessed treatment efficacy by OR (CR + PR) and toxicity by grade ≥3 AE. We calculated ORR and AE incidence via pooled data from the available studies and quantitative synthesis using the Stata metaprop procedure^15^; the patients without a measurable response were excluded from ORR analysis. Since individual‐level data were not available, we explored the relationship of treatment response between salvage nivolumab and ipilimumab and prior PD‐1/PD‐L1‐based therapy by the odds ratio of response. We assessed the between‐study heterogeneity by using the Cochran *Q* test and between‐group heterogeneity by using the *Z* test. A conservative random effect model was used to combine values from the different studies. All *p* values were two‐sided, and a value of less than 0.05 was considered statistically significant. All analyses were performed using the Stata software (StataCorp, College Station, TX, USA http://www.stata.com). Additionally, we performed the risk of bias assessment for the included studies using a validated tool for nonrandomized studies known as RoBANS as described,^16^ which contains six domains, including the selection of participants, confounding variables, the measurement of exposure, the blinding of the outcome assessments, incomplete outcome data, and selective outcome reporting.

## 3 RESULTS

### 3.1 Patient characteristics from OSU

As shown in Tabel S1, 27 patients with clear cell mRCC were included, with a median age of 61.4 years old. The patient numbers based on IMDC scores were 6, 13, and 5 for low‐, intermediate‐, and high‐risk stratification, whereas 3 patients were not classified due to missing information. Twenty‐three patients had prior nephrectomy. Based on prior treatment history, 8 patients had exposure to both PD‐1/PD‐L1 inhibitor and TKI, 18 patients had exposure to PD‐1 inhibitor alone, and 1 patient had exposure to PD‐1 inhibitor and high‐dose interleukin‐2 (IL‐2). Overall, 22 patients had a measurable response for ORR analysis, while all patients were available for PFS and toxicity analysis. A Kaplan–Meier survival curve for PFS was shown in Figure S2. Details of the analysis results were shown in Tables 1 and 2.

**TABLE 2:** Incidence of grade ≥3 adverse events

	Gul	Choueiri	Atkins	Yang	McKay	Sum	Pooled Analysis	Quan Synthesis	Checkmate214
*n*, incidence (*N*, sample size)	8 (45)	13 (46)	12 (30)	5 (27)	17 (57)	55 (205)	0.27 (0.21–0.33)	0.26 (0.19–0.34)	0.47 (0.42–0.51)
Liver	3	1	1	0	2	7	0.03 (0.01–0.07)		0.09 (0.06–0.11)
Diarrhea/colitis	2	5	4	2	3	16	0.08 (0.05–0.12)		0.05 (0.03–0.07)
Skin rash/pruritus	1	1	2	0	0	4	0.02 (0.01–0.05)		0.04 (0.02–0.06)
Kidney damage	0	0	2	0	0	2	0.01 (0–0.03)		0.01 (0.01–0.03)
Lung	1	0	1	2	2	6	0.03 (0.01–0.06)		0.01 (0–0.02)
Endocrinopathy	0	0	1	0	9	10	0.05 (0.02–0.09)		0.07 (0.05–0.09)
Fatigue	0	1	2	0	0	3	0.01 (0–0.04)		0.04 (0.03–0.06)
Lipase/amylase	0	3	7	0	0	10	0.05 (0.02–0.09)		0.1 (0.08–0.13)
Other	1	2	1	1	1	6	0.03 (0.01–0.06)		0.05 (0.03–0.07)

### 3.2 Study characteristics

The seven studies were grouped into two categories based on the study design (Table 1). In the standard group, following progressive disease (PD) on any prior line of treatment, patients received standard nivolumab and ipilimumab treatment (nivolumab and ipilimumab × 4 then nivolumab maintenance). Seventy‐nine percent of the patients received two or more lines of prior therapy, while 71% of the patients received VEGFR‐TKI either alone or in combination with an anti‐PD‐1/PD‐L1 antibody priorly (Table S2). In the adaptive group, patients received first‐line nivolumab monotherapy. Ipilimumab was added to nivolumab for stable disease (SD) or PD. The number of doses of ipilimumab ranged from 2 to 4 (4 for Atkins et al., 2 for McKay et al., and 2–4 for Grimm et al.). Nivolumab was continued until the second PD. All studies included patients of three IMDC risk groups, except the study by Grimm et al., which included only intermediate‐/high‐risk patients. An assessment of the bias of the included studies was summarized in Table S3.

### 3.3 Objective response

**FIGURE 1:**
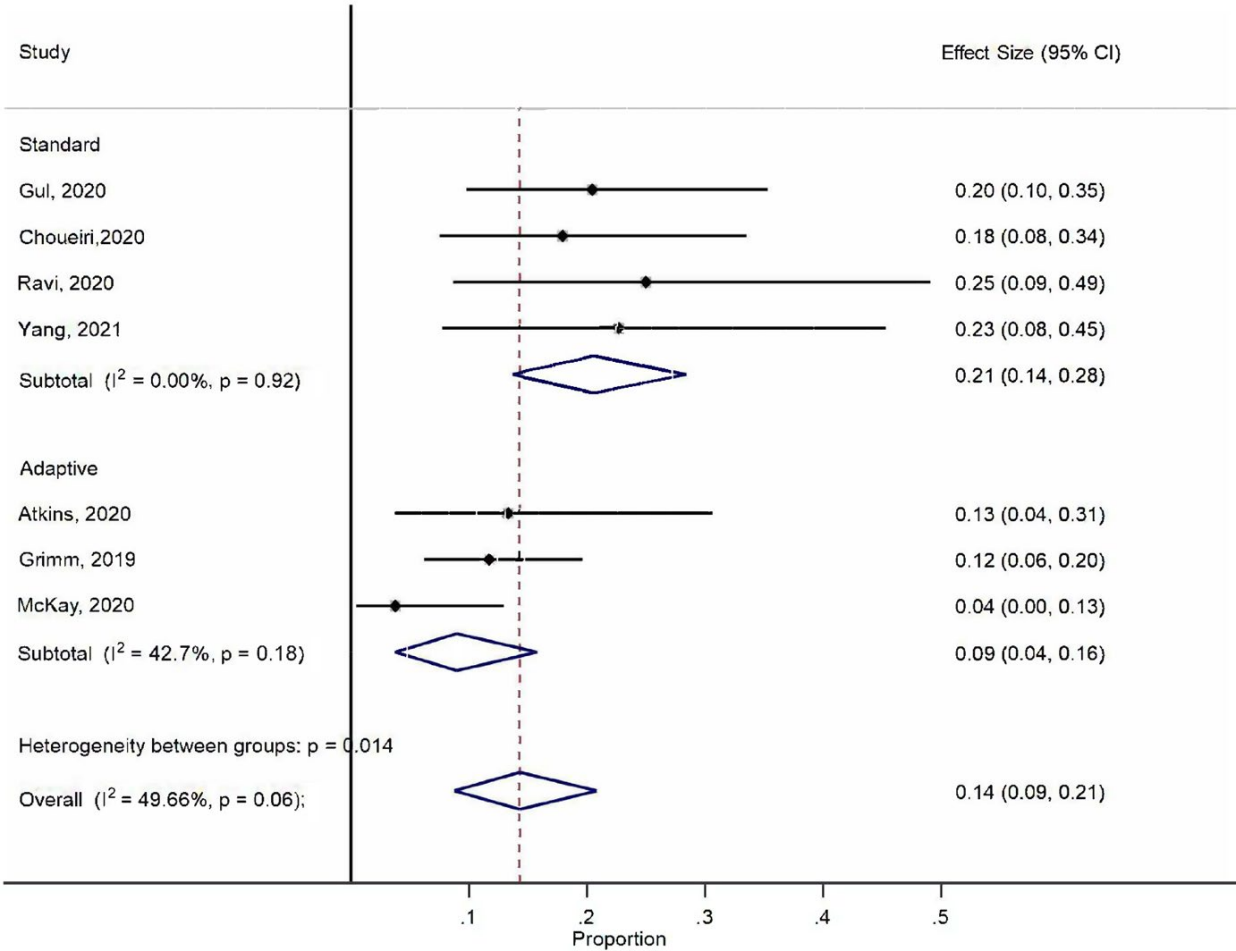
Quantitative synthesis of objective response rates for salvage nivolumab and ipilimumab treatment

**FIGURE 2:**
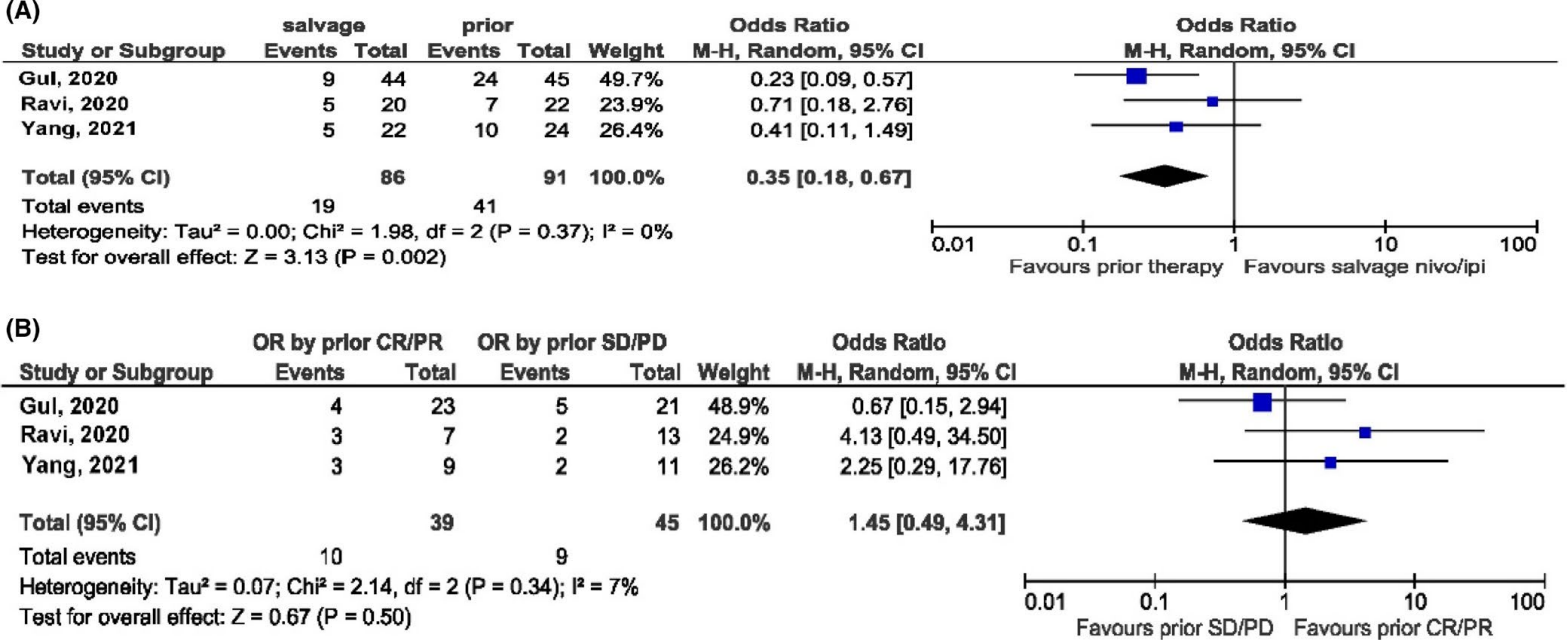
Explore the relationship of objective response rate between salvage nivolumab and ipilimumab and prior PD1/PD‐L1 inhibition. (A) Salvage vs. prior, (B) salvage by prior

In the standard group, 125 patients had evaluable responses, including 26 OR (1 CR and 25 PR). In the adaptive group, 18 out of 185 patients developed OR (4 CR and 14 PR). The pooled analysis and quantitative synthesis showed the ORR of 21% and 9–10% in the two groups, respectively. ORR was 14% when the two groups were combined (Table 1 and Figure 1). We further compared and correlated objective responses of salvage nivolumab and ipilimumab with prior ICI responses. The best overall response (BOR) to prior anti‐PD‐1/PD‐L1 therapy was available for three studies with a quantitative synthesis ORR of 44% (95% CI, 0.31–0.56). The ORR of salvage nivolumab and ipilimumab was significantly lower than that of prior anti‐PD‐1/PD‐L1 therapy (odds ratio = 0.35, 95% CI, 0.18–0.67; *p* < 0.01) (Figure 2A). The BOR to prior anti‐PD‐1/PD‐L1 therapy did not correlate with the salvage nivolumab and ipilimumab responses (odds ratio = 1.45, 95% CI, 0.49–4.31; *p* = 0.5) (Figure 2B) but seemed to favor a higher chance of response to salvage treatment if an objective response to prior anti‐PD‐1/PD‐L1 therapy was documented.

### 3.4 Progression‐free survival

The median PFS was available for five studies, ranging from 3.7 to 5.5 months (Table 1). Quantitative synthesis was not performed due to a lack of individual patient data for the majority of studies. We were unable to evaluate the duration of response due to a lack of data. But it is noteworthy that some of the responses were durable. In the OSU cohort, 60% (3/5) of the responders (including 1 CR) had PFS > 6 months, whereas it was 56% (5/9) in the Gu et al. study.

### 3.5 Toxicity

**FIGURE 3:**
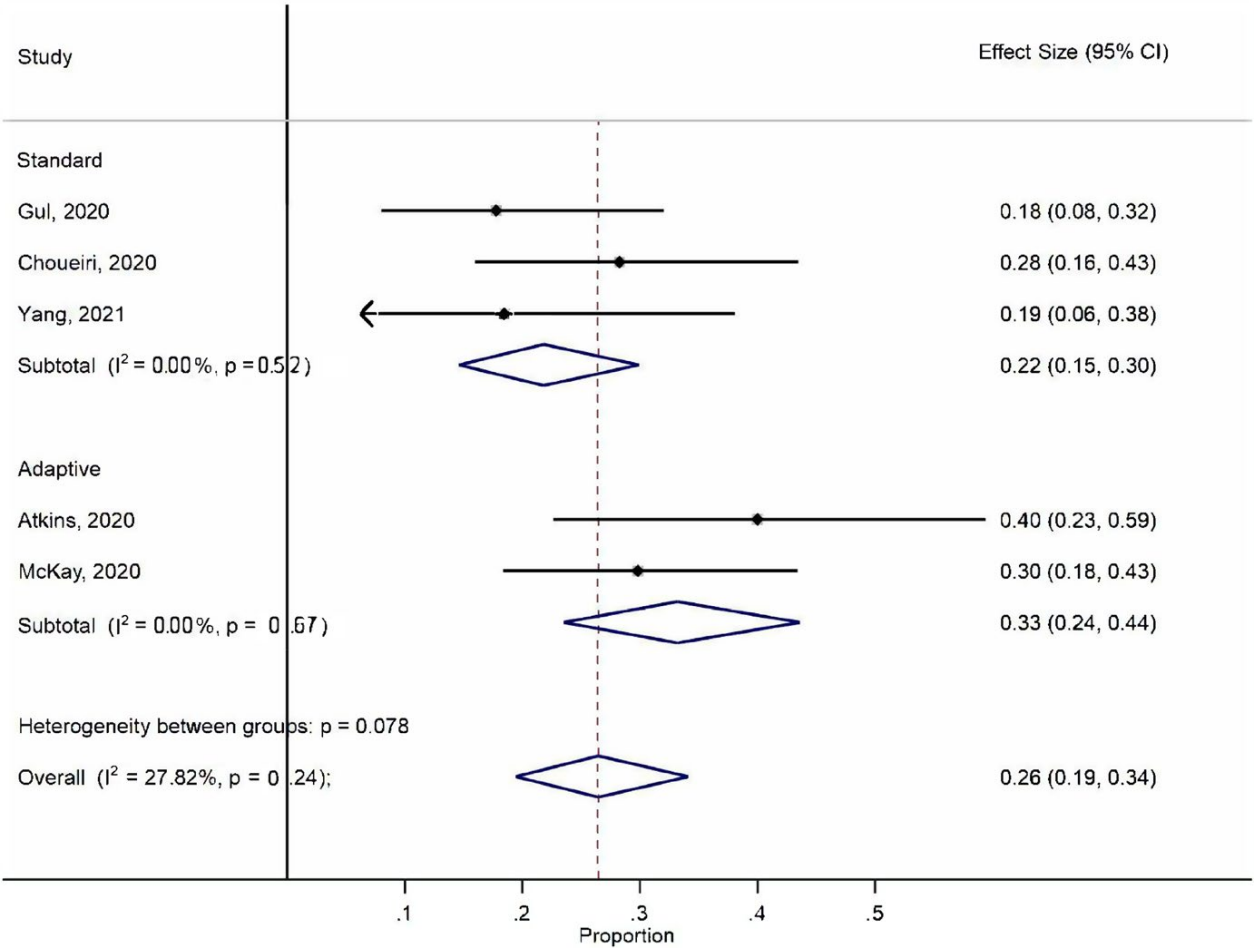
Quantitative synthesis of grade ≥3 adverse events for salvage nivolumab and ipilimumab treatment

Toxicity data were available for five studies (Table 2 and Figure 3). Fifty‐five out of 205 patients developed grade ≥3 AEs, yielding a pooled incidence of 27% (95% CI, 0.21–0.33). The most common grade ≥3 AEs were diarrhea/colitis (8%), endocrinopathy (5%), and elevated lipase/amylase (5%). We summarized grade ≥3 AEs reported in the Checkmate‐214 trial for comparison. The grade ≥3 AEs rate was numerically lower for salvage nivolumab and ipilimumab than front‐line, which was likely due to selection bias. No new safety signal was observed.

## 4 DISCUSSION

Nivolumab and ipilimumab are used widely in patients with mRCC, either in the first‐line or salvage settings. Accurate estimation of salvage nivolumab and ipilimumab treatment outcomes after prior anti‐PD‐1/PD‐L1 therapy is highly clinically relevant and may assist oncologists in decision‐making and treatment sequencing. Our study showed that the ORR of salvage nivolumab and ipilimumab was about 14% with a median PFS of around 4 months. However, treatment response seemed to differ between the adaptive design and the standard design.

It is plausible that most patients who had prior exposure to anti‐PD‐1/PD‐L1 therapy may not respond well to salvage nivolumab and ipilimumab. However, because of different mechanisms of action, ipilimumab may overcome the resistance to PD‐1/PD‐L1 inhibitors.^17^ Ipilimumab is an anti‐CTLA‐4 antibody promoting T cell priming by blocking the CTLA4‐B7 inhibitory signal at T cell and antigen‐presenting cell interface in the draining lymph nodes. Unlike ipilimumab, anti‐PD‐1/PD‐L1 antibodies work primarily by restoring the antitumor activity of preexisting T effector cells in the tumor microenvironment (TME). A primary anti‐PD1‐resistance mechanism is the lack of sufficient T cell infiltration.^18^ Ipilimumab may induce synergy by promoting tumor‐specific CD8^+^ T cell proliferation. In addition, other immune‐suppressive factors may also contribute to anti‐PD‐1 resistance, such as a high regulatory T cell to CD8^+^ T cell ratio. Ipilimumab can deplete CTLA‐4‐positive regulatory T cells through antibody‐dependent cell‐mediated cytotoxicity.^19^ Therefore, ipilimumab may resensitize tumors to nivolumab by promoting T cell priming and reducing T regulatory cells in ipilimumab naive patients. In contrast, nivolumab and ipilimumab rechallenge showed little efficacy in patients who already acquired resistance to both anti‐PD‐1 and anti‐CTLA4 antibodies.^20^

We classified the studies into standard and adaptive groups because of different designs and heterogeneity between groups (*p* = 0.014 by *Z* test). It is noteworthy to point out that our meta‐analysis included an independent patient cohort from OSU, which was not published before. The details of the patient characteristics were described in the manuscript. Overall, the treatment response and toxicity results were similar to other studies of the same category (standard design).

The three studies using the adaptive method enrolled patients in the first‐line setting who were treatment‐naive. After primary resistance to nivolumab monotherapy (SD or PD), these patients received an immediate boost by adding ipilimumab. In contrast, the standard studies included patients who were heavily pretreated. Indeed, some patients received salvage nivolumab and ipilimumab beyond the fourth line (Table S2), which may negatively impact salvage nivolumab and ipilimumab efficacy. However, compared with adaptive treatment, the ORR seemed to favor standard nivolumab and ipilimumab treatment in the salvage setting (21% vs. 9–10%). This observation may be explained by escape mechanisms rising from treatment pressure that is different from primary resistance. Many patients in the standard design may not be intrinsically resistant to anti‐PD‐1 antibodies but may have developed acquired resistance over time.^21^ In addition, exposure to VEGFR‐TKI was allowed in the standard studies. VEGFR‐TKIs can induce immunomodulatory effects on TME by enhancing the antitumor activity of CD8^+^ T cells and depleting myeloid‐derived suppressor cells.^22^ VEGFR‐TKI may also eradicate some ICI‐resistant tumor clones through its different antitumor mechanisms. Alternatively, it is possible that some patients included in the standard design stopped prior anti‐PD1/PD‐L1 regimens due to adverse events, which may contribute to a higher ORR by salvage nivolumab and ipilimumab later on. Unfortunately, we were unable to perform in‐depth analyses in this regard because the majority of the studies did not report such data. Nevertheless, in our cohort (Yang 2021), the majority (80%) of the salvage responders discontinued prior anti‐PD1/PD‐L1 therapy after disease progression. Only one patient stopped prior nivolumab due to toxicity who tolerated salvage nivolumab and ipilimumab well and achieved a complete response. Overall, our meta‐analysis suggests that salvage nivolumab and ipilimumab treatment is feasible. Treatment of standard design produces a moderately significant response, but the adaptive design is not the optimal approach.

As expected, our results showed that the ORR of salvage nivolumab and ipilimumab was lower, compared with its use in the first‐line setting (42%).^7^ It was also lower, compared with the ORR of prior ICI therapy in the same patient population (odds ratio = 0.35, 95% CI, 0.18–0.67). Although our results did not support the use of the response to prior ICI to predict the response to salvage nivolumab and ipilimumab therapy, it could be due to the inadequate statistical power from small sample sizes. In our study, quantitative synthesis of PFS was not possible due to a lack of individual data. However, all five studies showed a consistent median PFS of around 4 months of salvage nivolumab and ipilimumab (extrapolating median PFS to be about 4.6 months when 1 L and 2 L are combined in the study of Grimm et al). This number is smaller than what we observed in the first‐line setting (about 9.7 months).^7^ Whether more doses of ipilimumab would prolong the PFS is unclear. The long‐term use of ipilimumab is often limited by toxicity.^23^ The incidence of grade ≥3 AEs was only about 27%, half of that observed in the first‐line setting of the Checkmate‐214 trial. It is likely due to a screening bias from patient selection for ICI retreatment and recall bias of retrospective studies. The rate of grade ≥3 AEs was similar in the standard and adaptive groups.

It will be interesting to compare the efficacy of other salvage therapies with the efficacy of salvage nivolumab and ipilimumab after prior anti‐PD‐1/PD‐L1 therapy. Second‐line TKI treatment after failure of first‐line nivolumab and ipilimumab showed an ORR of 36% and a median PFS of 7–8 months.^24^ The ORR of TKI after nivolumab was 27% with a median PFS of 8.9 months in a separate retrospective study.^25^ The efficacy of salvage ICI/TKI in ICI‐pretreated patients was reported in a Phase II study, which demonstrated an ORR of 55.8% and a median PFS of 12.2 months by pembrolizumab and lenvatinib.^26^ Although some individual patients might achieve relatively longer PFS, both ORR and PFS seem to fall short by salvage nivolumab and ipilimumab therapy, compared with TKI‐incorporated therapies. This is probably because TKI inhibits mRCC by mechanisms independent of immunotherapy, hence avoiding the cross‐resistance from treatment with similar mechanisms.

Our study has several limitations. First, there was significant heterogeneity among the studies. Such variation was reduced by grouping the studies based on the designs. Second, information on patients who discontinued prior anti‐PD‐1 therapy due to AEs rather than progressive disease was unavailable. We speculate that they would represent a small percentage of the cohort since these patients were determined to be fit for salvage nivolumab and ipilimumab, unlikely to significantly impact our conclusion. Third, we cannot determine the detailed response correlation between prior and salvage ICI therapies due to a lack of individual data. Nevertheless, the odds ratio analysis is a measure of association between an exposure and an outcome and therefore is a valid method to infer the correlation. Fourth, our analysis was based mainly on data extracted directly from the published literature. It was subject to the quality of each study. As shown in Table S3, some of the studies had a high risk of bias in related domains and therefore their conclusions need to be taken with caution. Three out of the seven studies (Choueiri, Atkins, and Grimm) were only reported in abstract form, but more details of the three studies were available through presentations at national/international conferences. The reported clinical outcomes are unlikely to change before and after publication since the study methodology is fixed and data collection has been completed. Due to the sparse available evidence, we included these three studies in the analysis.^27^ Last, we did not present overall survival as a study endpoint because i) such analyses would be heavily biased due to heterogeneity of treatment and the timing of nivolumab and ipilimumab initiation and ii) few studies reported results of overall survival.

## 5 CONCLUSION

Salvage nivolumab and ipilimumab showed antitumor activity and a manageable safety profile in patients with mRCC who had prior anti‐PD‐1/PD‐L1 therapy, regardless of their prior response. This meta‐analysis enables more accurate extrapolation of existing efficacy and toxicity data regarding salvage nivolumab and ipilimumab with greater statistical power. As such, it furthers our understanding of the optimal management of this disease.

## DISCLOSURE

This work was presented as an abstract at the American Society of Genitourinary Cancers Symposium 2021.

## CONFLICT OF INTEREST

None of the contributing authors have any conflicts of interest, including specific financial interests and relationships and affiliations relevant to the subject matter or materials discussed in the manuscript.

## AUTHOR CONTRIBUTION

Conception and design: Ming Yin, Sherry V. Morri, Megan Hinkley. Acquisition of data: All authors. Analysis and interpretation of data: All authors. Drafting of the manuscript: Yuanquan Yang, Ming Yin, Sherry V. Morri. Critical revision of the manuscript: All authors. Statistical analysis: Ming Yin. Supervision: Ming Yin.

## ETHICS STATEMENT

Ethical approval was not sought from an institutional review board or ethics committee prior to commencing this study.

## Supporting information


Table S1–S3

Figure S1–S2

## Data Availability

All data generated or analyzed during this study are included in this published article and its supplementary information files.
