# Supplementary material for: Salvage nivolumab and ipilimumab after prior anti‐PD‐1/PD‐L1 therapy in metastatic renal cell carcinoma: A meta‐analysis
Source: Cancer Med. 2022 Feb 9;11(7):1669–77. doi: 10.1002/cam4.4587 (PMC8986145; doi:10.1002/cam4.4587)
Supplement: Supplementary file 1 — Table S1–S3 [file CAM4-11-1669-s001.docx]

| **Supplementary Table 1. OSU patient characteristics** | |
| --- | --- |
| **Parameters** | **No. (%)** |
| Median age (range) | 61.4 (22.9-83.3) |
| Gender |  |
| M | 17 |
| F | 10 |
| Histology |  |
| clear cell | 27 |
| IMDC risk group |  |
| Favorable | 6 |
| Intermediate | 13 |
| Poor | 5 |
| Unknown | 3 |
| Nephrectomy |  |
| Yes | 23 |
| No | 4 |
| Prior systemic therapy lines |  |
| 1 | 6 |
| 2 | 11 |
| 3 | 3 |
| ≥4 | 7 |
| Prior ICI therapy |  |
| PD-1/PD-L1 inhibitor + TKI | 8 |
| PD-1 inhibitor alone | 18 |
| PD-1 inhibitor + IL2 | 1 |

| **Supplementary Table 2. Lines of prior systemic therapy** | | | | | |  |
| --- | --- | --- | --- | --- | --- | --- |
| **Study** | **No. of prior line of systemic therapy** | | | | | **Patient No.** |
|  | **1** | **2** | **3** | **≥3** | **≥4** | **prior VEGFR TKI** |
| **Gul** | 9 | 12 | 8 |  | 16 | 27 |
| **Choueiri** | 10 | 12 |  | 23 |  | 37 |
| **Ravi** | NA | NA | NA | NA | NA | NA |
| **Yang** | 6 | 11 | 3 |  | 7 | 20 |
|  |  |  |  |  |  |  |

| **Supplementary Table 3.** **The risk of bias assessment by RoBANS tool** | | | | | | |  |
| --- | --- | --- | --- | --- | --- | --- | --- |
|  | **Gul** | **Choueiri** | **Ravi** | **Yang** | **Atkins** | **Grimm** | **McKay** |
| **The selection of participants** | High risk | Low risk | High risk | High risk | Low risk | Low risk | Low risk |
| **Confounding variables** | High risk | Low risk | High risk | High risk | Low risk | Low risk | Low risk |
| **Measurement of exposure** | Low risk | Low risk | Low risk | Low risk | Low risk | Low risk | Low risk |
| **Blinding of outcome assessments** | Low risk | Low risk | Low risk | Low risk | Low risk | Low risk | Low risk |
| **Incomplete outcome data** | Unclear risk | Low risk | Unclear risk | Unclear risk | High risk | Low risk | Low risk |
| **Selective outcome reporting** | Low risk | Low risk | Low risk | Low risk | Low risk | Low risk | Low risk |
