# Supplementary material for: Salvage nivolumab and ipilimumab after prior anti‐PD‐1/PD‐L1 therapy in metastatic renal cell carcinoma: A meta‐analysis
Source: Cancer Med. 2022 Feb 9;11(7):1669–77. doi: 10.1002/cam4.4587 (PMC8986145; doi:10.1002/cam4.4587)
Supplement: Supplementary file 2 — Figure S1–S2 [file CAM4-11-1669-s002.pptx]

## Slide 1
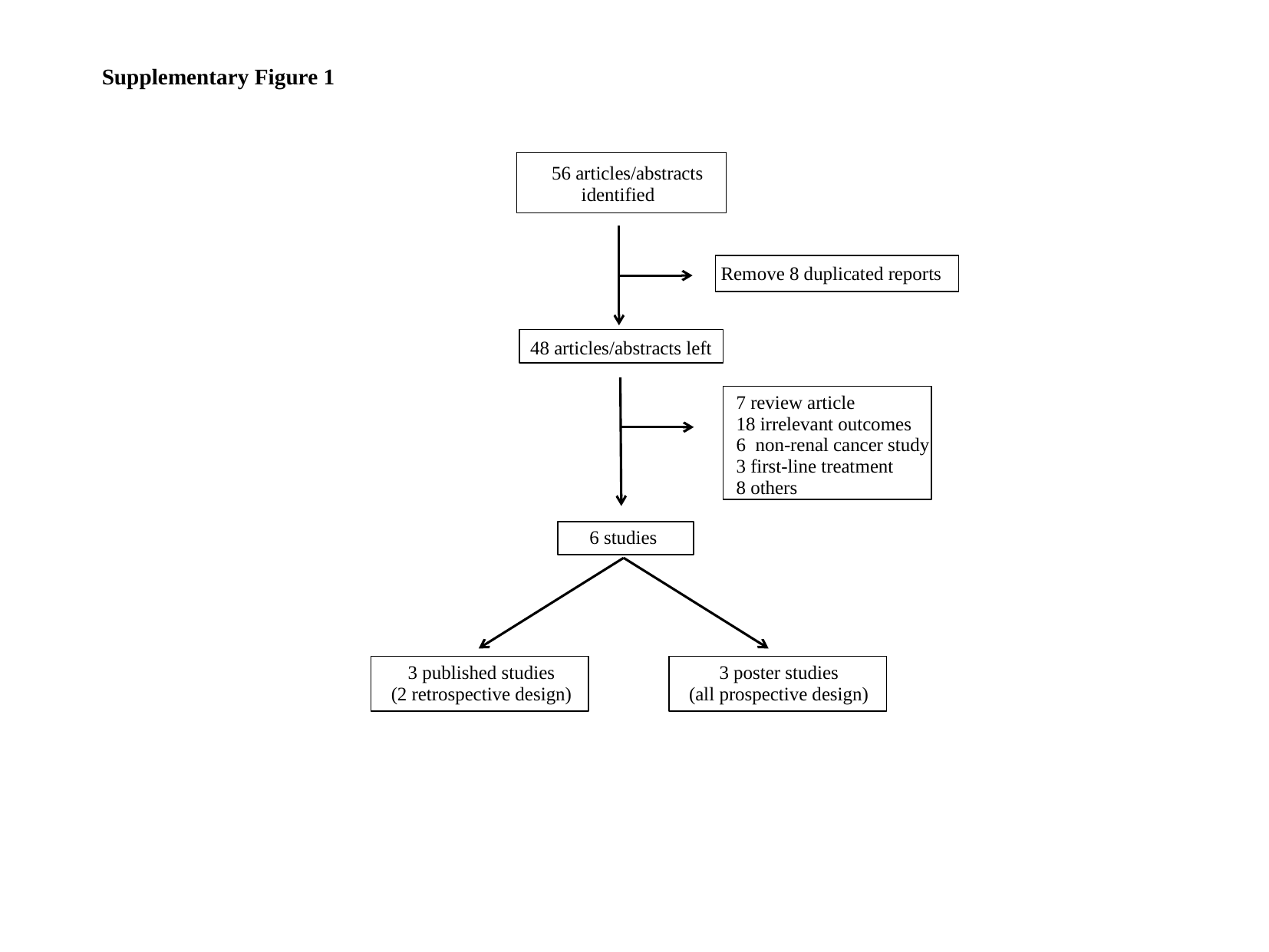

Supplementary Figure 1
 56 articles/abstracts identified
Remove 8 duplicated reports
 48 articles/abstracts left
 7 review article
 18 irrelevant outcomes
 6 non-renal cancer study
 3 first-line treatment
 8 others
 6 studies
3 published studies
(2 retrospective design)
3 poster studies
(all prospective design)

## Slide 2
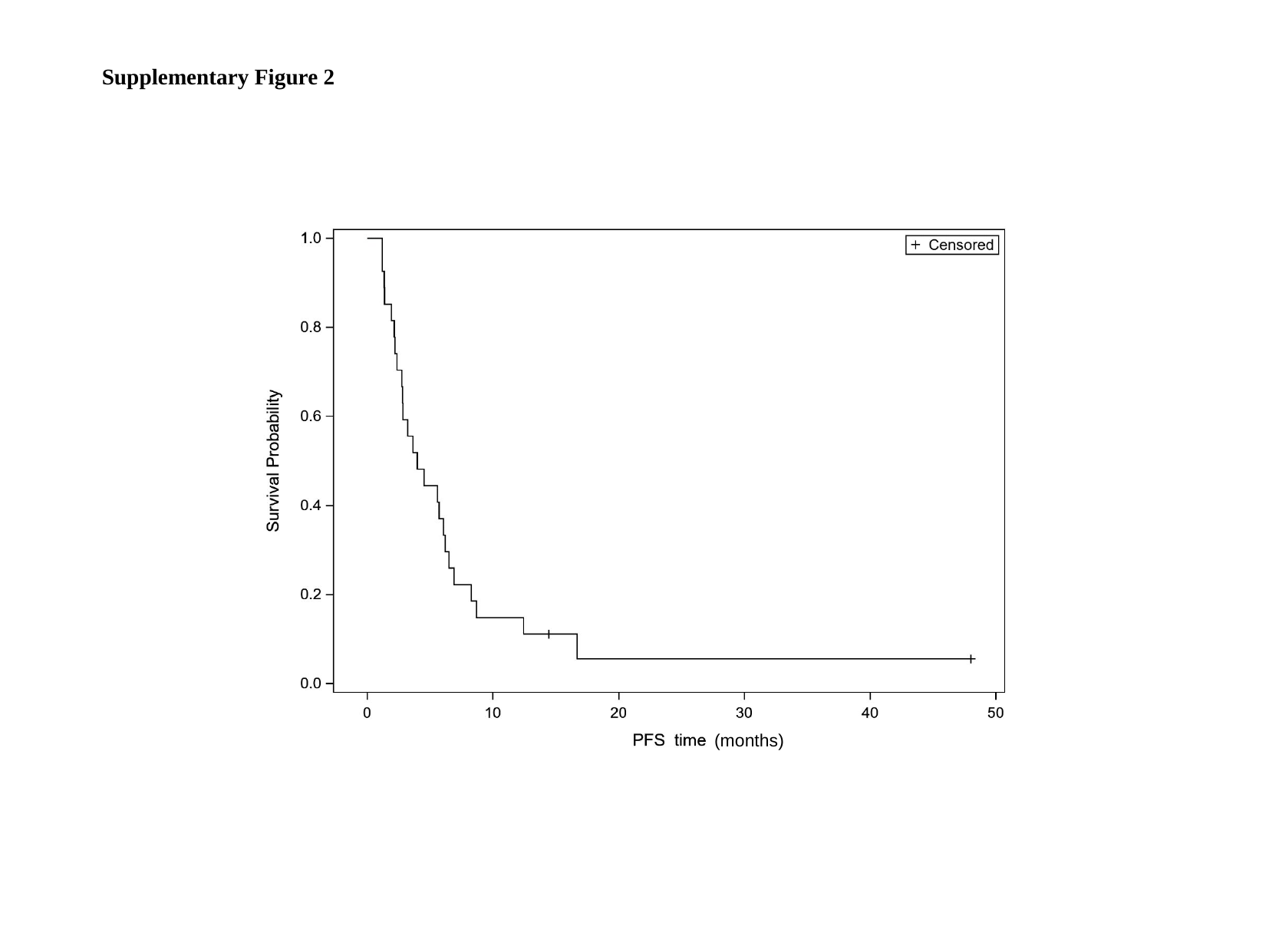

Supplementary Figure 2
(months)
